# Predictors of infective endocarditis associated in-hospital mortality in Ayder Comprehensive Specialized Hospital, Tigray, North Ethiopia: Microbiological,clinical features, and management profiles

**DOI:** 10.1371/journal.pone.0300322

**Published:** 2024-05-02

**Authors:** Hagazi Tesfay, Yemane Weldu, Mohamedawel Mohamedniguss Ebrahim, Abraha Hailu, Kibreab Gidey, Teklay Gebrehaweria, Samuel Berhane, Zekarias Gessesse, Hagos Kahsay, Daniel Mezmur, Kidan Fisseha, Aregawi Haileselassie, Alemayehu Bayray

**Affiliations:** 1 Department of Internal Medicine, School of Medicine, College of Health Sciences, Mekelle University, Mekelle, Tigray Region, Ethiopia; 2 Department of Medical Microbiology and Immunology, College of Health Sciences, Mekelle University, Mekelle, Tigray Region, Ethiopia; 3 School of Public Health, College of Health Sciences, Mekelle University, Mekelle, Tigray Region, Ethiopia; University of Catania: Universita degli Studi di Catania, ITALY

## Abstract

**Background:**

Infective endocarditis (IE) is a continuously evolving disease with a high mortality rate despite different advances in treatment. In Ethiopia, there is a paucity of data regarding IE. Therefore, this study is aimed at assessing IE-related in-hospital mortality and characterization of IE patients based on their microbiological, clinical features, and management profiles in the Ayder Comprehensive Specified Hospital (ACSH).

**Methods:**

We conducted a hospital-based prospective follow-up study with all consecutive sampling techniques for suspected infective endocarditis patients admitted to ACSH from January 2020 to February 2022. Echocardiography was performed, and three sets of blood samples for blood culture were taken as per the standard protocol. We also performed isolation of microbial etiologies and antimicrobial susceptibility tests. The data was analyzed using STATA version 16. Stepwise logistic regression was run to identify predictors of in-hospital mortality. Effects were measured through the odds ratio at the 5% level of significance.

**Results:**

Seventy-four cases of suspected infective endocarditis were investigated; of these, 54 episodes fulfilled modified Duke’s criteria. Rheumatic heart disease (RHD) (85.2%) was the most common underlying heart disease. Murmur (94.4%), fever (68.5%), and pallor (57.4%) were the most common clinical findings. Vegetation was present in 96.3% of episodes. Blood culture was positive only in 7 (13%) episodes. Complications occurred in 41 (75.9%) cases, with congestive heart failure being the most common. All patients were managed medically, with no surgical intervention. The in-hospital mortality was 14 (25.9%). IE-related in-hospital mortality was significantly associated with surgery recommendation and myalgia clinical symptoms.

**Conclusion:**

IE occurred relatively in a younger population, with RHD as the most common underlying heart disease. There was a high rate of culture-negative endocarditis, and the majority of patients were treated empirically. Mortality was high. The establishment of cardiac surgery and strengthening microbiology services should be given top priority.

## Background

Infective Endocarditis (IE) is an infection of the endocardial lining of the heart with pre-existing lesions or on intra-cardiac prosthetic device [1–4]. The crude incidence of IE ranged between 1.5 and 11.6 cases per 100,000 people [5]. Bacterial species such as *Staphylococcus* and *Streptococcus* account for 80% of cases; however, it may occasionally be due to fungal pathogens as well [1].

Infective endocarditis may present with very different clinical patterns depending on organs involved, the underlying cardiac disease, the micro-organisms involved, the presence or absence of complications, and the patient’s characteristics [6, 7]. The systematic diagnosis of IE is based on the Duke Classification that stratifies patients with suspected IE into three categories: definite, possible, and rejected IE. The Duke Classification is mainly based on major criteria like the results of blood cultures and either trans thoracic echocardiography (TTE) or trans esophageal echocardiography (TEE) and minor criteria [6, 7]. Echocardiography as the main diagnostic tool in endocarditis is recommended as the first-line imaging modality in suspected IE [6–10]. Additionally, most important laboratory investigation in the diagnosis of IE is blood culture. Studies have shown that there is a continuous bacteremia and high rate of positive blood culture in infective endocarditis. In the absence of recent prior antimicrobial therapy, up to 95% of culture positive results are reported for causative microorganism [11]. In developing countries, studies have shown blood culture negative endocarditis is in the range of 40–90% [12–15].

IE remains an important cause of morbidity and mortality in all age groups and it is one of the most feared complications of structural heart lesions [1, 5, 8, 13, 16]. Death rate due to IE, as high as 22.6% has been reported from sub-Saharan Africa [12]. Several independent predictors of in-hospital mortality were mentioned in the literature [8, 13, 14, 17, 18]. Africa carries high burden of rheumatic heart disease, which carries a risk of IE and congestive heart failure. As in many parts of sub Saharan Africa and particularly in Ethiopia, RHD is one of the leading causes of hospital admissions due to heart failure. The most important precipitant factors are infections like pneumonia and infective endocarditis [4]. Although historically rheumatic valvulitis was considered a frequent predisposing factor for endocarditis, time has changed, mitral valve prolapse, aortic sclerosis, and bicuspid aortic valvular heart disease are now more frequent causes in developed countries [19]. The emerging population at risk for IE consists of patients with health care-associated infections, elderly patients with valvular sclerosis, patients with valvular prostheses, and hemodialysis patients [20].

The continuous change in the clinical profile of the disease mandates regular appraisal of the management strategies. Thus, despite several guidelines available to treat the disease IE has always been a challenge for the physicians [21]. In Ethiopia and other sub-Saharan African countries, where most health facilities are understaffed, the diagnosis and treatment of IE can be a big challenge and it is likely for cases of IE to be missed [4].

Our experience shows that microbiological support in the diagnosis of IE in our hospital and other parts of Ethiopia is low and positive blood culture in suspected cases of IE is pretty low. This circumstance encouraged us to study the characteristics of Ethiopian patients who presented with a suspected diagnosis of IE. Moreover, in Ethiopia, particularly in the study area, there is scarcity of data on infective endocarditis in adults. Therefore, this study aimed to assess the IE related in-hospital mortality and characterization of IE patients based on their microbiological, clinical features and management profiles in Ayder Comprehensive Specialized Hospital, Northern Ethiopia.

## Materials and methods

### Study setting

The study was conducted in Ayder Comprehensive Specialized Hospital (ACSH), located in Mekelle, Tigray 783 kilometers north of Addis Ababa, Capital of Ethiopia. The hospital commenced rendering its referral and specialized medical services in 2008 to a population of around 10 million in its catchment areas of the Tigray, Afar and North-eastern parts of the Amhara regional states. ACSH provides a broad range of medical services to both in and out patients in its main departments including Internal medicine, Surgery, Gynecology and obstetrics, Pediatrics, Radiology, Pathology, Dermatology among others. It stands as the second largest hospital in the nation with the total capacity of about 500 inpatient beds in the four major departments and other specialty units along with eight other affiliated hospitals in the Tigray region. The department of internal medicine has 108 beds in its inpatient department with Pulmonology, Gastroenterology, Neurology and Nephrology services equipped with bronchoscopies, upper and lower Gastrointestinal Endoscopies, Electroencephalography and dialysis setups. The cardiology unit has 5 cardiologists and is equipped with VIVID E-9 echocardiography machine, modern resting and treadmill stress electrocardiogram machines, Holter-Electrocardiograms, Holter Blood Pressure monitor services as well as Catheterization laboratory.

### Study design, study period and inclusion criteria

A hospital based prospective follow up study design was conducted from January 2020 to February 2022. All adult patients suspected with IE and admitted to Ayder comprehensive specialized hospital for further diagnosis and management were included in the current study.

### Sample size determination and sampling technique

To recruit participants suspected with IE, all consecutive sampling approach was applied. Thus only 74 participants suspected with IE were recruited in two years’ period. Of which, 54 had definitive or possible diagnosis with IE according to the modified duke’s criteria.

### Study variables

#### Dependent variable

The dependent variable was IE associated treatment outcome which is labelled as 1 if patient had died and 0 otherwise (recovered/survived).

#### Independent variables

In this study the independent variables were: age, sex, address, prior antibiotic use, type of antibiotic taken and its duration, presence of recent medical procedures and cardiac operations, previous history of infective endocarditis, clinical and laboratory profile, duration of illness, underlying heart lesion and presence of risk factors, presence of coexisting disease such as diabetes mellitus, alcohol abuse, smoking, HIV/AIDS, intravenous drug use, haemodialysis history, vegetation on echocardiography: site and size, bacteriology, type of antibiotic regimen, surgery and its indications, and duration of hospital stay.

### Data collection methods and procedures

A data collection tool (structured questionnaire) was developed to obtain data including socio- demographics, previous history and risk factors for IE, clinical findings, and result of requested investigations, treatment, complications and outcome. The socio- demographics, previous history and risk factors for IE part was filled by trained Ward Nurses while clinical findings, and result of requested investigations, treatment, complications and outcome part was filled by trained Medical Residents. Diagnosis was made based on modified Dukes criteria, which proposed two major, one major+3 minor or five minor criteria. Patients were classified as definite, possible and rejected cases. Definite and possible cases were considered as Infective endocarditis and further analysed.

All cardiac patients who presented to the hospital with suspected infective endocarditis were included. Examination and clinical findings were recorded and all patients had three sets of blood culture at least one hour apart within 24 hours.

Culture result was considered typical if classical organisms like *Streptococci*, *Staphylococci*, *Enterococci* or HACEK group were found. A single isolation of coagulase negative *Staphylococci* were considered contamination. Complete blood count, erythrocyte sedimentation rate, renal function, rheumatoid factor, urine for microscopic haematuria and chest x-ray were requested for all patients. Other blood tests like electrolytes, liver function test and serology were requested when necessary and indicated.

Transthoracic echocardiography was performed by experienced cardiologists. Vegetation was defined typical if a mobile dense mass is found attached to the valve or their supporting structure. Suspicious echo cardiac densities not meeting the major criteria were classified as minor criteria.

Abdominal ultrasound was performed when complications such as renal, splenic infarction or abscess were suspected. Brain computed tomography scan was performed when neurological complications occurred. When the diagnosis is made, empirical treatment was started with ceftriaxone 1 gm IV BID or penicillin group 2–3 million I.V every 4 hours + gentamicin; 1mg/kg q 8 hours for the first two weeks and then penicillin or ceftriaxone for the last two weeks for a total of 28 days. The drugs were renal adjusted when necessary. In cases of acute IE or critically ill patients, vancomycin was added. Antibiotic modification was done upon arrival of the culture and sensitivity results. Patients were followed until discharge, and information regarding antibiotic dosage, duration, medical therapy outcomes, complications, and indications for surgery were recorded.

### Specimen collection and transport

Three 10 ml blood samples were collected from each patient at an interval of at least 1 hour over a period of 24 hours. The collected specimen were inoculated into blood culture bottles containing base broth and incubated at 37°c. Brain heart infusion broth was used with the following ingredients per 100 c.c. of the media: Bacto yeast extract 5gms, Para-aminobenzoic acid 0.05 g, sucrose 300 g, Thioglycollic acid 3x10^5^ M, L-cysteine 10^4^ M. Hundred ml of this broth was poured into each blood culture bottle to form a layer of about 8 cm deep. Para-aminobenzoic acid to counteract the effect of sulphonamides, sucrose as an osmotic stabilizer, thyglycollin acid and L-cysteine as reducing agent were used.

### Processing of blood samples

#### Culture and biochemical tests

Bacterial colonies were initially characterized by morphology and microscopic examination. Then, colonies were sub-cultured onto mannitol salt agar and 5% Sheep’s blood agar (Oxoid, UK). Further identification was done by biochemical tests using the standard bacteriological techniques.

#### Antimicrobial susceptibility testing

The antimicrobial susceptibility testing was done on Mueller-Hinton agar (Oxoid, UK) for every potential pathogenic bacterial isolates with the common antibiotics by Kirby-Bauer disk diffusion method matching the test organism to 0.5 McFarland turbidity standards. Then, the susceptibility result was interpreted according to the principles established by Clinical and Laboratory Standards Institute (CLSI) by measuring the zone diameter of inhibition. Prior to application, antibiotic discs potency was checked by using *Staphylococcus aureus* ATCC25923, *Escherichia coli* ATCC 25922 and *Pseudomonas aeruginosa* ATCC 27853 strains as control organisms.

### Quality control

Reagents and materials were carefully selected from reputable sources to ensure high quality. Standard operating procedures were followed with quality control measures during processing of the specimens. Culture Media were prepared based on the manufacturers’ instructions. Sterility of the culture media was checked by incubating 5% of the batch at 37°C. After overnight incubation, culture media which showed bacterial growth were discarded. Quality of the culture media and antimicrobial susceptibility was checked using standard reference strains of *Staphylococcus aureus* ATCC25923, *Escherichia coli* ATCC 25922 and *Pseudomonas aeruginosa* ATCC 27853.

### Operational definitions

#### Early Prosthetic Valve Endocarditis (PVE)

IE that occurred within 1 year of surgical implantation of the prosthesis. Whereas IE that occurred afterwards was considered late PVE.

#### Health-Care Associated IE (HAE)

Was defined as (1) nosocomial IE: IE occurred ≥ 48 hrs after hospital admission. (2) Non nosocomial IE: IE occurring within the first 48 hours of hospitalization in a patient exposed to health-care procedures (a) 1 month of receiving IV cannulation, chemotherapy, or dialysis; (b) 3 months after admission into an acute care facility; or (c) any time after admission into a nursing home and 5) invasive procedure and surgery within the year prior to infection.

#### Community-acquired IE

IE diagnosed upon admission (or within the first 48 hours of hospitalization) in a patient that has not been exposed to care procedures.

#### Intravenous Drug Users (IVDU)-associated IE

If the patient reported injection drug use within 3 months of the onset of IE symptoms.

### Data entry and analysis

Data were collected and entered into Epi Data 4.6 and then exported to STATA version 16 for data management, cleaning, and statistical analysis. Both descriptive and inferential statistical methods were employed. Continuous variables were described using mean and standard deviation or median and interquartile range depending on the distribution of data. Categorical variables were described using frequency and percentage. Chi-square assumption was checked using cross-tabulation and for variables that violate the assumption–their categories with few cell counts were merged together. The sample size is relatively small to run all more than ten candidate variables simultaneously. Therefore, Stepwise logistic regression with forward selection method was run to identify independent variables that adequately predict IE associated death. Model selection and model comparison was done using likelihood ratio test to select a robust model that best describes the data well. According to the likelihood ratio test statistic, the logistic regression model which includes five variables (surgery recommendation, myalgia, renal failure, vegetation site, and severity of pulmonary hypertension) is superior over the null and other reduced models. Collinearity diagnostics was checked using variance inflation factor and none of the variables in the final model had collinearity problems. Eventually, the effects of the explanatory variables on the odds of IE associated in-hospital mortality was measured through odds ratio (OR) and its corresponding confidence intervals.

### Ethical considerations

Ethical approval letter was solicited from Institutional Review Board of the College of Health Sciences, Mekelle University and the ethical approval number is: ERC 13175/2020. Written informed consent was obtained from each study participant. Test results were delivered to participants with proper treatment for individuals with positive test results. Integrity of patient’s data, the privacy and the confidentiality of disease control data were ensured by strictly protecting unauthorized disclosure of personal identifying information.

## Results

### Socio-demographic characteristics

A total of 74 participants were investigated for infective endocarditis, of these, 54 (73%) fulfilled the modified Duke’s criteria as Definite IE (36), Possible IE (18) cases while 20 participants were rejected. The reasons for rejection were: alternative diagnosis reached 11 (55%), fever disappeared within 4 days of treatment 6 (30%) and did not fulfill definite or possible IE 3 (15%). Native and prosthetic valves were involved in 52 (96.3%) and 2 (3.7%) patients respectively.

Age of the participants with infective endocarditis (definite or possible) ranged 18 to 86 years. The median age of the study participants was 27 years. More than half (53.7%) of the participants were males. The majority (68.5%) of the patients resided in urban areas and 75.9% of the participants had basic reading and writing skills. About one third (27.8%) of the participants were students, while farmers accounted for 20.4% of the cases **[Table 1].**

**Table 1:** Socio-demographic characteristics of IE (Definite or possible) patients in Ayder Comprehensive Specialized Hospital, Tigray region, Northern Ethiopia, 2020–2022 (n = 54).

Socio-demographic variables	Category	Count (%)	IE status	
			Definite (%)	Possible (%)
**Age in years—Median (IQR)**		27 (24.37)	27 (24–33.5	28.5 (21–45)
**Sex**	Male	29 (53.7)	17 (58.6)	12 (41.4)
	Female	25 (46.3)	19 (76.0)	6 (24)
**Occupation**	Housewife	11 (20.4)	6 (54.6)	5 (45.4)
	Farmer	11 (20.4)	8 (72.7)	3 (27.3)
	Merchant	5 (9.3)	4 (80.0)	1 (20.0)
	Student	15 (27.8)	9 (60.0)	6 (40.0)
	Government employee	4 (7.4)	3 (75.0)	1 (25.0)
	Other (unemployed/driver…)	8 (14.8)	6 (75.0)	2 (25.0)
**Place of residence**	Urban	37 (68.5)	25 (67.6)	12 (32.4)
	Rural	17 (31.5)	11 (64.7)	6 (35.3)
**Education**	Illiterate	13 (24.1)	9 (69.2)	3 (30.8)
	Literate	41 (75.9)	27 (65.8)	14 (34.2)

IQR = Interquartile Range

### Previous history and risk factors

Of the 54 (definite or possible) cases of infective endocarditis, 9 (16.7%) had previous history of infective endocarditis. Recent medical procedures were done in 16 (29.6%) of the cases. Intravenous catheterizations 6 (37.5%), Genitourinary (GU) 4 (25.0%) and Dental procedures 4 (25.0%) were recorded. Three of the GU procedures were abortion related. The majority (70.4%) of cases were admitted without definite presumed source.

Twenty-three patients (42.6%) had taken antibiotics prior to admission to our hospital (before blood was drawn for blood culture). Ceftriaxone was given for 15 (27.8%) cases, metronidazole in 3 cases, ciprofloxacin and azithromycin in 2 cases. Antibiotics were given for mean duration of 5 days. Three patients had previous cardiac operations, two valve replacements and one pacemaker lead insertion. There were no IV drug users and hemodialysis cases. Overall, 15 patients (27.8%) had history of admissions in the last 3 months prior to symptoms. Co-morbidities were seen in 9 (16.7%) participants [**Table 2].**

**Table 2:** Previous history and risk factors of IE (Definite or possible) patients in Ayder Comprehensive Specialized Hospital, Tigray region, Northern Ethiopia, 2020–2022 (n = 54).

Previous history and risk factors	Category	Count (%)	IE status	
			Definite (%)	Possible (%)
**Previous history of IE**	Yes	9 (16.7)	4 (44.4)	5 (55.6)
	No	65 (83.3)	32 (71.1)	13 (28.9)
**Admission in health facility in the past 3 months prior to symptoms**	Yes	15 (27.8)	12 (80.0)	3 (20.0)
	No	39 (72.2)	24 (61.5)	24 (38.5)
**If yes, facility type (n = 15)**	Hospital	14 (93.3)	12 (85.7)	2 (12.3)
	Health center	1 (6.7)	0 (0.0)	1 (100.0)
**Recent Medical procedures**	Yes	16 (29.6)	3 (18.8)	13 (81.2)
	No	38 (70.4)	28 (63.6)	16 (36.4)
**Type of medical procedure (n = 16)**	Intravenous catheters	6 (37.5)	5 (83.3)	1 (16.7)
	Dental procedures	4 (25.0)	3 (75.0)	1 (25.0)
	Genitourinary procedures	4 (25.0)	4 (100.0)	0 (0.0)
	Pace maker pocket abscess drainage	1 (6.3)	1 (100.0)	0 (0.0)
	Chest tube insertion	1 (6.3)	0 (0.0)	1 (100.0)
**Prior antibiotic use (<2wks before admission)**	Yes	23 (42.6)	14 (60.9)	9 (39.1)
	No	31(57.4)	22 (71.0)	9 (29.0)
**Previous cardiac operation (n = 23)**	< = 7	17 (73.9)	9 (52.9)	8 (47.1)
	>7	6 (26.1)	5 (83.3)	1 (16.7)
**Previous cardiac operation**	Yes	3 (5.6)	1 (33.3)	2 (66.7)
	No	51 (94.4)	35 (68.6)	16 (31.37)
**Comorbidities (HIV, TB, HTN, DM, or Epilepsy)**	Yes	9 (16.7)	7 (77.8)	2 (22.2)
	No	45 (83.3)	29 (64.4)	16 (35.6)

HIV: human immunodeficiency virus; TB: tuberculosis; HTN: hypertension; DM: diabetes mellitus

### Clinical features

Fever (87%), weakness (87%), shortness of breath (85.2%) were commonly reported symptoms. Fever was the presenting symptom in 87% of cases. The median duration of the presenting symptom prior to diagnosis was 14 days, suggesting late presentation. However, fever was objectively documented in 68.5%. Heart murmur was seen in 94.4% while new murmur was detected in 33.3% of the cases. Pallor accounted for 57.4% and splenomegaly occurred in 25.9%. About 16.7% were noted to have an acute neurological deficit, and other peripheral signs were rare findings **[Table 3].**

**Table 3:** Clinical features of infective endocarditis (Definite or possible) patients in Ayder Comprehensive Specialized Hospital, Tigray region, Northern Ethiopia, 2020–2022 (n = 54).

Variable	Category	Count (%)	IE status	
			Definite (%)	Possible (%)
**Duration of illness in days (Median and IQR)**			14 (7–28)	
**Fever**	Yes	47 (87.0)	35 (74.5)	12 (25.5)
	No	7 (13.0)	1 (14.3)	6 (85.7)
**Chills**	Yes	37 (68.5)	27 (73.0)	10 (27.0)
	No	17 (31.5)	9 (52.9)	8 (47.1)
**Weakness**	Yes	47 (87.0)	32 (68.1)	15 (21.9)
	No	7 (13.0)	4 (57.1)	3 (42.9)
**Malaise**	Yes	41 (75.9)	29 (70.7)	12 (29.3)
	No	13 (24.1)	7 (53.8)	6 (42.2)
**Anorexia**	Yes	39 (72.2)	25 (64.1)	14 (35.9)
	No	15 (27.8)	11 (73.3)	4 (26.7)
**Shortness of breath**	Yes	46 (85.2)	28 (60.9)	18 (39.1)
	No	8 (14.8)	8 (100.0)	0 (0.00)
**Cough**	Yes	42 (77.8)	28 (66.7)	14 (33.3)
	No	12 (22.2)	8 (66.7)	4 (33.3)
**Sweating**	Yes	28 (51.8)	20 (71.4)	8 (28.6)
	No	26 (48.2)	16 (61.5)	10 (38.5)
**Arthralgia**	Yes	17 (31.5)	11 (64.7)	6 (35.3)
	No	37 (68.5)	25 (67.6)	12 (32.4)
**Headache**	Yes	28 (51.8)	19 (67.9)	9 (32.1)
	No	26 (48.2)	17 (65.4)	9 (34.6)
**Weight loss**	Yes	29 (53.7)	19 (65.5)	10 (34.5)
	No	25 (46.3)	17 (68.0)	8 932.0)
**Myalgia**	Yes	20 (37.0)	16 (80.0)	4 (20.0)
	No	34 (63.0)	20 (58.8)	14 (41.2)
**Stroke symptoms**	Yes	12 (22.2)	11 (91.7)	1 (8.3)
	No	42 (77.8)	25 (59.5)	17 (40.5)
**Confusion/delirium**	Yes	11 (20.4)	8 (72.7)	3 (27.3)
	No	43 (79.6)	28 (65.1)	15 (34.9)
**Nausea/Vomiting**	Yes	22 (40.7)	15 (68.2)	7 (31.8)
	No	32 (59.3)	21 (65.6)	11 (34.4)
**Edema**	Yes	36 (66.7)	24 (66.7)	12 (33.3)
	No	18 (33.3)	12 (66.7)	6 (33.3)
**Chest pain**	Yes	20 (37.0)	11(55.0)	9 (45.0)
	No	34 (63.0)	25 (73.5)	9 (26.5)
**Abdominal pain**	Yes	14 (25.9)	10 (71.4)	4 (28.9)
	No	40 (74.1)	26 (65.0)	14 (35.0)
**Hemoptysis**	Yes	7 (13.0)	5 (71.4)	2 (28.6)
	No	47 (87.0)	31 (66.0)	16 (34.0)
**Back pain**	Yes	11 (20.4)	5 (45.5)	6 (54.5)
	No	43 (79.6)	31 (72.1)	12 (27.9)
**Hematuria**	Yes	32 (59.3)	23 (71.9)	9 (28.1)
	No	22 (40.7)	13 (59.1)	9 (40.9)
**Signs**				
**Fever (Temperature >38°C)**	Yes	37 (68.5)	31 (83.8)	6 (16.2)
	No	17 (31.5)	5 (29.4)	12 (70.6)
**Pallor**	Yes	31 (57.4)	24 (77.4)	7 (22.6)
	No	23 (42.6)	12 (52.2)	11 (47.8)
**Heart murmur**	Yes	51 (94.4)	34 (66.7)	17 (33.3)
	No	3 (5.6)	2 (66.7)	1 (33.3)
**New murmur (n = 51)**	Yes	17 (66.7)	10 (58.8)	7 (41.2)
	No	34 (33.3)	24 (70.6)	10 (29.4)
**Splenomegaly**	Yes	14 (25.9)	12 (85.7)	2 (14.3)
	No	40 (74.1)	24 (60.0)	16 (40.0)
**Hepatomegaly**	Yes	13 (24.1)	6 (46.2)	7 (53.8)
	No	41 (75.9)	30 (73.2)	11 (26.8)
**Splinter hemorrhage**	Yes	1 (1.9)	1 (100.0)	0 (0.0)
	No	53 (98.1)	35 (66.0)	18 (34.0)
**Finger clubbing**	Yes	13 (24.1)	11 (84.6)	2 (15.4)
	No	41 (75.9)	25 (61.0)	16 (39.0)
**Jane way lesion**	Yes	1 (1.9)	1 (100.0)	0 (0.0)
	No	53 (98.1)	35 (66.0)	18 (34.0)
**Convulsion**	Yes	3 (5.6)	3 (100.0)	0 (0.0)
	No	51 (94.4)	33 (64.7)	18 (35.3)
**Neck stiffness**	Yes	3 (5.6)	3 (100.0)	0 (0.0)
	No	51 (94.4)	33 (64.7)	18 (35.3)
**Vascular embolic event**	Yes	9 (16.7)	8 (88.9)	1 (11.1)
	No	45 (83.3)	28 (62.2)	17 (37.8)

### Laboratory and microbiology findings

On routine investigations, White Blood Cell (WBC) count >12,000 and <4000 were reported in 12 (22.2%) and 8 (14.8%) respectively. Elevated creatinine was seen in 33.3% and 11.1% had baseline serum creatinine more than two. Erythrocyte Sedimentation Rate (ESR) was raised (>30 mm/hr) in 87% of the patients. Significant anemia (Hemoglobin level <10g/dl) was present in 42.6% of cases, while 3.7% had hemoglobin below 7 g/dl. Hematuria and proteinuria were present in 66.7%. Rheumatoid factor was reactive in 22.2% of the participants.

Blood culture was positive in only 7 (13%) cases; gram-negative bacteria were the prevailing organisms representing 57.1% of positive blood culture isolates: which includes 2 *Klebsiella* species, 1 *Pseudomonas aeruginosa* and 1 *Escherichia coli* isolates. Other bacterial isolates were *Streptococcus pyogenes* (1 isolate), 1 *Staphylococcus aureus* and 1 *Staphylococcus epidermidis* **[Table 4]**.

**Table 4:** Laboratory and microbiology profiles of IE (Definite or possible) patients in Ayder Comprehensive Specialized Hospital, Tigray region, Northern Ethiopia, 2020–2022 (n = 54).

Variable	Category	Count (%)	IE status	
			Definite (%)	Possible (%)
**Hemoglobin level (g/dl)**	<10 g/dl	23 (42.6)	13 (73.9)	6 (26.1)
	> = 10 g/dl	31 (57.4)	19 (62.3)	12 (38.7)
**WBC count (cells/mm^3^)**	<4000	8 (14.8)	5 (62.5)	3 (37.5)
	4000–12000	34 (63.0)	24 (70.6)	10 (29.4)
	>12000	12 (22.2)	7(58.3)	5 (41.7)
**ESR in mm/hr**	1–30	7 (13.0)	3 (42.9)	4 (57.1)
	31–100	37 (68.5)	24 (64.9)	13 (35.1)
	> = 100	10 (18.5)	9 (90.0)	1 (100.0)
**Serum creatinine level (mg/dl)**	< = 1.1	36 (66.7)	24 (66.7)	12 (33.3)
	>1.1	18 (33.3)	12 (66.7)	6 (66.7)
**Rheumatoid factor**	Negative	42 (77.8)	11 (91.7)	1 (8.3)
	Positive	12 (22.2)	25 (59.5)	17 (40.5)
**Microscopic hematuria**	Yes	36 (66.7)	29 (80.6)	7 (19.4)
	No	18 (33.3)	7 (38.9)	11 (61.1)
**Blood culture**	Negative	47 (87.0)	31 (66.0)	16 (34.0)
	Positive	7 (13.0)	5 (71.4)	2 (28.6)
**Bacterial isolates (n = 7)**	*Staphylococcus aureus*	1 (14.3)	1 (100.0)	0 (0.0)
	Coagulase negative *Staphylococcus*	1 (14.3)	1 (100.0)	0 (0.0)
	*Escherichia coli*	1 (14.3)	1 (100.0)	0 (0.0)
	*Klebsiella ozaenae*	1 (14.3)	1 (100.0)	0 (0.0)
	*Klebsiella pneumoniae*	1 (14.3)	1 (100.0)	0 (0.0)
	*Pseudomonas aeruginosa*	1 (14.3)	1 (100.0)	0 (0.0)
	*Streptococcus pyogenes*	1 (14.3)	1 (100.0)	0 (0.0)

### Antibiotic sensitivity

Concerning antibiotic sensitivity, tetracycline, ampicillin, and erythromycin faced more resistance compared to other antibiotics. *Staphylococcus aureus* was sensitive to ceftriaxone, cloxacillin and ciprofloxacin but was resistant to erythromycin and tetracycline. *Pseudomonas aeruginosa* was sensitive to ciprofloxacin and gentamicin. Other gram negative bacilli had variable sensitivity but almost all except *pseudomonas* were sensitive to ceftriaxone and chloramphenicol **[Table 5]**.

**Table 5:** Antibiotic sensitivity of the identified organisms (n = 7).

	Type of isolate						
Type of antibiotic	*Staphylococcus aureus*	Coagulase negative *Staphylococcus*	*Escherichia coli*	*Klebsiella ozaenae*	*Klebsiella pneumoniae*	*Pseudomonas aeruginosa*	*Streptococcus pyogenes*
Ampicillin			S	R	R	R	S
Amoxicillin/Clavulanic acid	S						
Azithromycin					R		
Cefotaxime	S		S				
Ceftriaxone	S		S	S			
Chloramphenicol	S		S	S	S	R	S
Ciprofloxacin	S			R	S	S	
Cotrimoxazole	S		S	S	R	R	
Cloxacillin	S						
Clindamycin	S						S
Erythromycin	R						R
Gentamycin	S	S				S	
Imipenem					S	R	
Oxacillin	S	S					
Penicillin		S					
Tetracycline	R		R	R	R		
Vancomycin		S					S

S: Sensitive, R: Resistant

### Echocardiography profile

All patients underwent TTE while TEE was performed in only 5 (9.3%) episodes. Vegetations were detected in 52 (96.3%) episodes. Mitral valve was involved in 30 (55.6%), aortic valve 18 (33.5%), both valves involved 1 (1.9%), tricuspid valve 1 (1.9%), pulmonary valve 1 (1.9%) and Ventricular Septal Defect (VSD) in 1 (1.9%) episodes. Vegetation of < 10 mm, 10–20 mm and >20mm size were 35 (67.3%), 16 (30.8%) and 1 (1.9%) respectively. Forty-six (85.2%) of the episodes were RHD of which pure regurgitations were observed in 13 (28.3%) episodes, one case with pure stenosis and majority 32 (69.6%) had combined regurgitation and stenosis. Overall, 80.4% of the RHD patients had severe disease.

Degenerative Valvular Heart Disease (DVHD) was observed in 3 cases and Congenital Heart Disease (CHD) was seen in 2 cases (VSD and Congenital Pulmonary Stenosis), pacemaker lead in Right Ventricle predisposed for one patient. Both the PVIE occurred after 1 year of the valve replacement. Cardiac abscess was not detected but chordal rupture (1 episodes), cusp perforation (1 episode). Severe Pulmonary Hypertension (Pulmonary arterial systolic pressure >70mmHg) was observed in 20 (37%) episodes.

Left Ventricular Ejection Fraction (LVEF) ranged 15 to 77% with a mean of 61.1± 11.2%. Majority (85.2%, n = 46) had preserved LV systolic function with LVEF of >50% **[Table 6].**

**Table 6:** Echocardiographic profile of IE (Definite or possible) patients in Ayder Comprehensive Specialized Hospital, Tigray region, Northern Ethiopia, 2020–2022 (n = 54).

Variable	Category	Count (%)	IE status	
			Definite (%)	Possible (%)
**Vegetation present**	Yes	52 (96.3)	35 (67.3)	17 (32.7)
	No	2 (3.7)	1 (50.0)	1 (50.0)
**Vegetation size in mm (n = 52)**	<10 mm	35 (67.3)	22 (62.9)	13 (37.)
	10–20 mm	16 (30.8)	12 (75.0)	4 (25.0)
	>20 mm	1 (1.9)	1 (100.0)	0 (0.0)
**Vegetation mobility (n = 52)**	Mobile	52 (100.0)	35 (67.3)	17 (32.7)
	Fixed	0 (0.0)	0 (0.0)	0 (0.0)
**Vegetation site (n = 52)**	Mitral	30 (57.7)	22 (73.3)	8 (26.7)
	Aortic	18 (34.6)	12 (66.7)	6 (33.3)
	Tricuspid	1 (1.9)	1 (100.0	0 (0.0)
	Pulmonary	1 (1.9)	0 (0.0)	1 (100.0
	VSD	1 (1.9)	0 (0.0)	1 (100.0
	Mitral and aortic	1 (1.9)	0 (0.0)	1 (100.0
**Predisposing heart disease**	Yes	54 (100.0)	36 (66.7)	18 (33.3)
	No	0 (0.0)	0 (0.0)	0 (0.0)
**Type of predisposing heart disease**	RHD	46 (85.2)	35 (76.1)	11 (23.9)
	DVHD	3 (5.6)	0 (0.0)	3 (100)
	CHD	2 (3.7)	0 (0.0)	2 (100.0)
	PVIE	2 (3.7)	1 (50.0)	1 (50)
	Pacemaker lead	1 (1.9)	0 (0.0)	1 (100.0)
**RHD lesion type**	Regurgitation	13 (28.6)	12 (92.3)	1 (7.7)
	Stenosis	1(2.2)	1 (100.0)	0 (0.0)
	Combined	32 (69.6)	22 (68.8)	10 (31.2)
**RHD Severity**	Severe	37 (80.4)	27 (73.0)	10 (27.0)
	Non-Severe	9 (19.6)	8 (88.9)	1 (11.1)
**Severity of Pulmonary Hypertension**	Severe	20 (37.0)	12(60.0)	8 (40.0)
	Non-Severe	34 (63.0)	24 (70.6)	10 (29.4)
**Left ventricle ejection fraction (LVEF)**	< = 50%	8 (14.8)	4 (50.0)	4 (50.0)
	>50%	46 (85.2)	32 (69.6)	14 (30.4)
**Setting**	Health care associated	9 (16.7)	7 (77.8)	2 (22.2)
	Community acquired	45 (83.3)	29 64.4)	16 (35.6)
**Health care associated setting (n = 9)**	Nosocomial	4 (44.4)	4 (100.0)	0 (0.0)
	Non-nosocomial	5 (55.6)	3 (60.0)	2 (40.0)

VSD: Ventricular Septal Defect; RHD: Rheumatic Heart Disease; DVHD: Degenerative Valvular Heart Disease; CHD: Congenital Heart Disease; PVIE: Pulmonary valve infective endocarditis; LVEF: Left Ventricle Ejection Fraction.

### Complications

Complications were observed in 41 (75.9%) of IE patients. Congestive Heart Failure (CHF) was the most common (82.9%) complication observed followed by sepsis which occurred in 58.5% of the cases. Renal functions were deranged in 13 (31.7%), of which 5 cases underwent hemodialysis. Two patients were diagnosed gentamicin toxicity. Embolization occurred in 12 (29.3%) cases and majority 10 (83.3%) were cerebral emboli. Peripheral embolization to extremities occurred in 1 (4.5%) episode and spleen 1 (4.5%) episode. Septic pulmonary infarction occurred in 4 (9.8%) episodes. All these patients had right sided endocarditis due to VSD, valvular Pulmonary Stenosis (PS), Patent Ductus Arteriosus (PDA) and pace maker lead related tricuspid valve endocarditis; one case for each. Four cases were complicated by new conduction abnormality.

Pericardial effusion was seen in 12 (29.3%) cases. Mild and moderate pericardial effusions were seen in 11 and 1 patients respectively; however, severe pericardial effusion or cardiac tamponade did not occur. Persistent positive blood culture was documented in 3 cases. Local complications occurred in two cases, one had mitral cusp perforation and the other had Chordea-Tendineae rupture. More complications occurred on definite IE than possible IE **[Table 7]**.

**Table 7:** Complications of IE (Definite or possible) patients in Ayder Comprehensive Specialized Hospital, Tigray region, Northern Ethiopia, 2020–2022 (n = 54).

Variable	Category	Count (%)	IE status	
			Definite (%)	Possible (%)
**Complication**	Yes	41 (75.9)	28 (68.3)	13 (31.71)
	No	13 (24.1)	8 (61.5)	5 (38.5)
**Embolization (n = 41)**	Yes	12 (29.3)	11 (91.7)	1 (8.3)
	No	29 (70.7)	17 (58.6)	12 (41.4)
**Renal failure /creatinine >2 mg/dl (n = 41)**	Yes	13 (31.7)	8 (61.5)	5 (38.5)
	No	28 (68.3)	20 (71.4)	8 (28.6)
**Need dialysis (n = 41)**	Yes	5 (22.2)	2 (40.0)	3 (60.0)
	No	36 (87.8)	26 (72.2)	10 (27.8)
**New congested heart failure**	Yes	34 (82.9)	22 (64.7)	12 (35.3)
	No	7 (17.1)	28 (68.3)	13 (31.7)
**Septic pulmonary infarcts (n = 41)**	Yes	4 (9.8)	3 (75.0)	1 (25.0)
	No	37 (90.2)	25 (67.6)	12 (32.4)
**New conduction abnormality (n = 41)**	Yes	4 (9.8)	3 (75.0)	1 (25.0)
	No	37 (90.2)	25 (67.6)	12 (32.4)
**Sepsis (n = 41)**	Yes	24 (58.5)	19 (79.2)	5 (20.8)
	No	17 (41.5)	9 (52.9)	8 (47.1)
**Persistent Positive blood cultures (n = 41)**	Yes	3 (7.3)	2 (66.7)	1 (33.3)
	No	38 (92.7)	26 (68.4)	12 (31.6)
**Pericardial effusion (n = 41)**	Yes	12 (29.3)	10 (83.3)	2 (16.7)
	No	29 (70.7)	18 (62.1)	11 (37.9)
**Local complications perforation or rupture (n = 41)**	Yes	2 (4.9)	2 (100.0)	0 (0.0)
	No	39 (95.1)	26 (66.7)	13 (33.3)

### Management and treatment outcomes of IE

All patients were treated with IV antibiotics. Fifty-one (94.4%) of them started antibiotics empirically. Two patients started therapy after arrival of culture and sensitivity results. One patient started empirically and later antibiotics were modified based on culture and sensitivity results. Ceftriaxone with or without gentamicin was the most 47 (87%) commonly used antibiotic followed by gentamicin 36 (66.7%). Other antibiotics used were vancomycin 30 (55.6%), crystalline penicillin 3 (5.6%), ceftazidime 6 (11.1%), ampicillin 3 (5.6%), and ciprofloxacin in 2 (3.7%) episodes. The majority (64.8%) of patients received a four-week treatment (median duration of treatment). One patient developed allergic reaction in response to penicillin and was treated with vancomycin and gentamicin in combination and two patients developed gentamicin induced nephrotoxicity of whom one is died due to the toxicity.

No cardiac surgery was done as the hospital does not have the setup and was not also possible to refer them to other hospitals; initially patients were not affording, later conflict was added in the region. However, Surgery was recommended in 15 (27.8%) of the cases. The major 9 (60%) indication for surgery was worsening/refractory CHF with severe valve disease. Large vegetation (>15mm) with embolism was indicated in 2 episodes, heart failure with recurrent embolization 1 case, persistent infection despite adequate antibiotic therapy 1 case and one patient demanded removal of his infected pacemaker lead and battery. On elective bases 80% of RHDs actually needed surgery for valve replacement.

The in-hospital mortality of IE was 14 (25.9%). Cardiogenic shock was more responsible cause of death (seen in 35.7% of the patients), severe sepsis with septic shock 2 (14.3%), and respiratory failure in 2 (14.3%) patients (COVID-19 in 1 and ARDS in 1). Others were acute renal failure and CNS rebreeding (1 patient each) **[Table 8].**

**Table 8:** Management and treatment outcome of IE (Definite or possible) patients in Ayder Comprehensive Specialized Hospital, Tigray region, Northern Ethiopia, 2020–2022 (n = 54).

Variable	Category	Count (%)	IE status	
			Definite (%)	Possible (%)
**Mode of treatment**	Empiric	51 (94.4)	33 (64.7)	18 (35.3)
	Culture & sensitivity based	3 (5.6)	3 (100.0)	0 (0.0)
**Treatment duration** **(Median = 4 weeks)**	< 4 weeks	15 (27.8)	9 (60.0)	6 (40.0)
	4 weeks	35 (64.8)	27 (69.2)	12 (30.8)
	4 weeks	4 (7.4)	25 (71.4)	10 (28.6)
**Surgery recommended**	Yes	15 (27.8)	8 (53.3)	7 (46.7)
	No	39 (72.2)	28 (71.8)	11 (28.2)
**Indication for surgery (n = 15)**	Large vegetation with embolism	2 (13.3)	2 (100.0)	0 (0.0)
	Refractory to treatment	1 (6.7)	0 (0.0)	1 (100.0)
	Heart failure, Recurrent embolization	1 (6.7)	1 (100.0)	0 (0.0)
	Heart failure with severe valve disease	9 (60.0)	4 (44.4)	5 (55.6)
	Large vegetation with severe valve disease	1 (6.7)	1 (100.0)	0 (0.0)
	Removal of Pace maker lead and battery	1 (6.7)	0 (0.0)	1 (100.0)
**Treatment outcome**	Improved/recovered	40 (74.1)	27 (67.5)	13 (32.5)
	Death	14 (25.9)	9 (64.3)	5 (35.7)
**Causes of death (n = 14)**	Refractory heart failure	2 (14.3)	1 (50.0)	1 (50.0)
	Renal failure	1 (7.1)	0 (0.0)	1 (100.0)
	Severe sepsis with septic shock	2 (14.3)	1 (50.0)	1 (50.0)
	Cardiogenic shock	3 (21.4)	2 (66.7)	1 (33.3)
	Others*	6 (42.9)	5 (83.3)	1 (16.7)

Others*: Fatal Arrhythmia (Ventricular)/Re-bleeding (intraventicular) /respiratory failure

### Predictors of IE associated in-hospital mortality

A stepwise logistic regression with forward selection method was used to identify predictors’ of IE associated in-hospital mortality. Eventually, only five variables (surgery recommendation, myalgia, renal failure, vegetation site, and severity of pulmonary hypertension) were included in the final logistic model. However, only the 1^st^ two (surgery recommendation and myalgia) found to be significant predictors of death.

Keeping the effect of other variables constant, IE patients who had not been recommended to undergo surgery (AOR = 0.025; 95% CI: 0.002–0.313), and had no myalgia clinical symptom (AOR = 0.070; 95% CI: 0.006–0.794) had lower odds of death compared to patients who were recommended to undergo surgery and who had myalgia symptoms respectively. Renal failure, vegetation site, and, severity of pulmonary hypertension were not significant predictors in the current study. However, these variables are found to be logically linked with the outcome of interest and thus retained in the model to interpret their estimates. Absence of renal failure complication, and no severe pulmonary hypertension reduced the odds of death while vegetation site other than mitral increased the odds of developing death **[Table 9].**

**Table 9:** Predictors of in-hospital mortality among patients with IE in Ayder Comprehensive Specialized Hospital (n = 54).

Variable	Category	Death (%)	COR [95% CI]	AOR [95% CI]	Sig.
**Surgery recommended**	Yes	10 (66.7)	1.000		0.004**
	No	4 (10.3)	0.057 (0.013, 0.254)	0.025 (0.002–0.313)	
**Myalgia**	Yes	10 (50.0)	1.000		0.032*
	No	4 (11.8)	0.133 (0.034, 0.521)	0.070 (0.006, 0.794)	
**Renal failure**	Yes	6 (46.2)	1.000		0.099
	No	8 (19.5)	0.283 (0.074, 1.076)	0.165 (0.019, 1.407)	
**Vegetation site**	Mitral	4 (13.3)	1.000		0.431
	Aortic/others	10 (45.5)	5.417 (1.410, 20.816	2.206 (0.308, 15.818)	
**Severity of Pulmonary hypertension**	Severe	9 (45.0)	1.000		0.123
	Non-severe	5 (14.7)	0.211 (0.058, 0.770)	0.166 (0.017, 1.631)	

COR: Crude Odds Ratio; AOR: Adjusted Odds Ratio

*: significant at 5% level of significance

**: significant at 1% level of significance; 1.000: reference category

## Discussion

We found that a higher proportion of IE patients were males (53.7%). This is comparable to what was reported in several studies from elsewhere [14, 17]. The median age in our study was 27 years in accordance with what was found in studies from developing countries; Yemen 28years [15] and Egypt 30 years [13]. Whereas, in high income countries like USA and Europe, endocarditis occurred in patients over the age of 60 years [22, 23]. In a study done in Italy the mean age was 65.7±16 (SD) [24].

The majority of cases in the present study (70.4%) did not have definite presumed source of infection, which is higher than what was reported from Egypt 54% [14] and lower than the East China 92.6% [25].

It is known that structural heart disease, especially valvular heart disease is well-established risk factor for IE. In our study, all patients had predisposing heart disease and 90% of patients with infective endocarditis had RHD. This is very high proportion even if, RHD was reported as the chief underlying heart disease for IE in other low-income and middle-income countries in the world; Yemen 53.3% [15], South Africa 76.6% [26]. This high proportion of RHD is due to the endemicity of RHD in these countries. The prevalence of RHD in Ethiopia is reported 19 cases per 1000 and this is among the highest report in the world and similarly the proportion of RHD was 41.1% in our region [27, 28]. In contrast, RHD is identified in only 3% of patients with IE in Italy, and RHD is no longer the commonest underlying heart disease in western countries [29]. Degenerative Valvular Heart Disease (DVHD) was not common in patients with IE in this study (5.6%), contrasting with the findings from high-income countries where it is the major underlying cardiac disease in native valve IE [29]. This finding is most likely due to the younger age of patients with infective endocarditis in our setup; with a median age of less than 30 years.

The clinical presentation of IE is highly variable and may present as an acute, subacute or chronic condition reflecting the variable causative microorganisms, underlying cardiac conditions and pre-existing co morbidities [3, 30]. In the present study, majority of our patients (87%) had subjective fever, and fever was the presenting symptom in 87% of cases. However, fever was objectively documented in 68.5%. Heart murmur was seen in 94.4% while new murmur was detected on 33.3% of the cases. Splenomegaly occurred in 25.5%, pallor accounted for 57.4%, embolic events in 16.7% and other peripheral signs were rare findings. This shows that the absence of documented fever cannot rule out the diagnosis and high index of suspicion should be made when a patient has fever, heart murmurs or embolic events. In a study done in Egypt, all patients had fever, 58% had dyspnea, 14% had neurologic signs and 80% had murmurs. Peripheral signs were uncommon [14].

Identification of causative microorganisms is crucial for an adapted and effective antimicrobial therapy. Unfortunately, our study showed that in majority (87%) of patients with infective endocarditis no microorganism was identified. This is similar to what was reported in Yemen 90.3% [15]. Contrary to the results of our study, studies from high income countries reported about 10–20% of patients show no growth in blood culture [22, 29]. Potential reasons for such a low blood culture yield in our study may include the frequent use of antibiotics before blood collection (45% of these patients had received antibiotics), limited resource for microbiology investigations, automated machines that detect growth and possibly inappropriate blood sampling procedures. Our study shows that the microbiology profile of IE in our hospital has a unique feature with gram-negative bacilli predominance, especially *Klebsiella* species, these organisms also dominated the blood culture results done in our hospital (ICU) [31]. In contrast *Streptococci* in low income countries and *Staphylococci* in high income countries are the most common cause of IE [22, 24, 29, 32–34]. The reason for the lower report of *Streptococci* and *Staphylococcus* species in our hospital could also be the higher use of antibiotics that can cover these organisms and miss the gram negatives. These findings highlight teaching our practitioners to take blood cultures before antibiotics are given for patients suspected of IE. There are only few studies on IE caused by gram negative bacilli. A study conducted in Slovakia compared 564 cases of non-Gram-negative endocarditis with 42 cases of Gram-negative endocarditis [35]. In a review done by Guillermo Cuervo, microbial like gram negative bacilli (2–5%) and fungal (<2%) are among infrequent causes of IE [36].

Laboratory features are supportive evidences and some of them like findings on urine analysis (hematuria and proteinuria), rheumatoid factor may be used as minor criteria. Our study showed, 87% of patients had raised ESR, 42.6% of them had anemia and about 67% have evidence of glomerulonephritis on urine study. These findings are congruent with other studies [15, 26]. Serologic tests for the diagnosis of IE could help for our setup as the culture yield is low. We believe that the microbiology diagnosis can be improved by implementing either serological testing or molecular methods in order to detect rare and fastidious bacteria and fungi that are difficult to grow.

Echocardiography is very crucial in establishing the diagnosis of IE and remains the main accurate imaging modality to identify endocardial lesions associated with IE. The sensitivity for the diagnosis of vegetation in native and prosthetic valves is 70% and 50% respectively for TTE, but 96% and 92% respectively for TOE. Specificity has been reported to be around 90% for both TTE and TOE [3, 7, 26, 37]. It was especially more important in our setup because of low culture positivity (only 16%) in our series. In our study, all patients had TTE and only 5 patients underwent TEE, 2 patients with prosthetic valves and 3 cases with native valves. The lower rate of TEE is explained partly by patients refuse for fear of intubation and partly because of the diagnosis was clear with TTE. Vegetation was seen in 96.3%, in 2 cases (3.7%) had non vegetation endocarditis with new onset of valvular regurgitation considered as a major criterion. Like many other studies, the most commonly affected valve was the mitral valve, followed by the aortic valve and then the tricuspid valve and pulmonary valve [13–15, 17, 25, 26, 38].

Our research study showed that the overall complication rate was high, with 75.9%, and this is similar with the findings of Cresti et al, who reported a complication rate of 77%, and Serra et al, who reported a rate of 60.7% [24, 33]. The high complication rate in our site could be our patients have advanced underlying rheumatic heart disease, late presentation and most importantly the absence of surgical services. Early surgery reduces complication as noted by different studies [33, 34]. The three main complications were Heart Failure(HF), sepsis, and renal failure in accordance with the study done by Fernandes et al [17]. Congestive heart failure (CHF) is a leading cause of death in IE patients. It is the most frequent complication of IE as well as most frequent indication for surgery in such cases. Valve destruction causing acute regurgitation is the most characteristic lesion leading to HF in native valve IE [22]. In the present study, CHF presented in 63% of the study patients and isolated valvular insufficiency was reported in 28.3% of HF cases. In the French study, 58.7% had CHF and it was the main indication for surgery [17]. M, Sadaka et al. reported CHF in 58% [14]. Yemeni study reported 37.5% and Chinese study 69.0%. In all studies, higher rate of valve regurgitation was reported [22, 25]. Sepsis was the second most common complication reported in our study (58. 5%). Renal impairment was seen in 31.7% of our cases, 5 cases (12.5%) needed hemodialysis. In a study from South Africa, acute kidney injury was noted in 21.9% of cases [18].

Embolic episodes are an important cause of morbidity and mortality. Dislodgements of part of vegetation of infected tissue or intra-cardiac thrombi may cause embolic events, the most common complication during IE. Embolic events are frequent and life-threatening complications of IE related to the migration of cardiac vegetation. In a prospective study of 50 patients done by Mohammed Sadaka, 26% of IE cases presented with different sites due to systemic embolization whereas a study from South Africa reported higher embolic complications (36.1%) [18]. In our current study, major embolic episodes occurred in 12 patients (29.3%) leading to 4 deaths (33.3%). The site of vegetation in mitral position was significant risk to life threatening embolization. Stroke was seen in 11 cases, 9 ischemic and 1 hemorrhagic. Regarding the peripheral emboli, one was to the spleen and the other one who had concomitant cerebral emboli was to the right brachial artery. It is important to mention that pericarditis is a very rare complication of endocarditis and its presence in a developing country should point more towards rheumatic or tuberculosis etiology. In the present study the higher proportion of mild pericardial effusions is possibly as part of systemic congestion in heart failure.

COVID-19 diagnosis may delay the recognition of more dramatic illnesses such as infective endocarditis, which is a dreaded complication in patients with cardiac disease. In two case report studies conducted by Amal El Ouarradi in Morocco, a 50-year-old female patient, followed for atrial fibrillation and rheumatic valvulopathy under anti vitamin K., died of heart failure and hemorrhagic stroke. Another case of 26-year-old patient with a history of surgical closure of an interventricularseptal defect at the age of 12 died of respiratory failure and shock [39]. In our study, we have an 18 years old female with RHD diagnosed with COVID pneumonia and later IE, died of ARDS despite proper antibiotics and steroid. Hence, COVID 19 had contributed to delay the diagnosis of IE.

Prompt antibiotic therapy can avoid the incidence of severe sepsis, multiple organ dysfunctions Syndrome, risk of stroke, and sudden death. Therefore, appropriate antibiotic therapy should be initiated as soon as possible after microbiological test in suspected or confirmed IE cases [3, 7, 37].

In our study, all patients were treated with IV antibiotics. Fifty-one (94.4%) of them started antibiotics empirically. Ceftriaxone and gentamicin were the most commonly used antibiotic accounting for 87% and 66.7% respectively. There was also a higher use of vancomycin (55.6%). Culture and sensitivity was only helpful for 3 patients.

Several studies have reported that surgery if done early for selected patients with complicated IE, reduces mortality, and embolic complications [25, 32, 33]. In our study 15% needed emergency cardiac operation for IE while 74% needed elective valve replacement. Unfortunately, our patients were denied of these because there was no cardiac surgical center in our region. In the latter half of the study, it was not possible even for referral as a result of the brutal war and Tigray siege. This also happens in some African studies, no surgical treatment was performed in a study done by Djibril Marie et al, while it has become an important adjunct to medical therapy in the management of IE [40]. A survey of cardiac surgery in Africa conducted in 2013 showed 1 cardiac surgeon for 14.3 million people in SSA, a cardiac center for 33 million in Africa while 1 center for 120,000 in USA [12]. More than 50% of patients with endocarditis will need valve surgery [29]. In a retrospective study of 174 Chinese patients by Huimin Xu, 43.7% of the patients underwent surgery leading to a lower mortality of 10.9% [25]. In another study conducted in India, cardiac surgery was done in 23.1% and more deaths could have been prevented by early cardiac surgery [38]. M. Sadaka et al from Alexandria hospital reported that surgery was done in 39% of his patients while 82% were actually indicated [14].

Despite advances in pharmaceutical and surgical treatment, IE remains a fatal disease [3, 7, 37] The in-hospital mortality for IE has not improved, and stroke, embolism, other than stroke, heart failure and other complications remain common [3, 7, 37]. The in hospital mortality is 25.9% in the present study that was higher than both developing and developed countries, systematic review in Africa: 22.6% (ranging 11.2–33.2%), high income countries 10–20% [24, 32–34]. Taking the in availability of surgical setup and conflict and siege in the region, the mortality rate is comparable. This is due to our patients are much younger with less co-morbidities other than their underlying heart disease. Because older age and with morbidity poses an increased risk of mortality in IE. Therapy under a multispecialty team including an infectious disease specialist, cardiologist, and cardiac surgeon has been reported to reduce the time to surgery and both operative and long-term mortality [36]. However, there is no such a team established in our Hospital. The present study showed that the odds of death were lower in patients who haven’t been recommended surgery as intervention and with no myalgia clinical symptom. A study conducted in Egypt showed that any surgical indication was among the most significant independent predictors of in-hospital mortality [13].

### Limitation of the study

One of the limitations of our study is that it was conducted in a single-center tertiary hospital, and hence the findings cannot be generalized to the community or to lower-level health care facilities. A substantial referral bias may be observed in hospital-based studies. The other limitations are the small sample size and the absence of serologic tests for selected organisms. Due to the outbreak of COVID-19 and later the brutal war that broke out in November 2020 and the subsequent siege in the region, it was not possible to buy the reagents from abroad. Finally, long-term follow-up of IE patients and long-term mortality rate assessment were not possible. Despite limitations, this study provides valuable data on the clinical characteristics of IE in our region and possibly contributes to the improvement of the clinical management of IE in our hospital. In addition, it will alert policymakers to consider having a cardiac surgery center because we believe that mortality could have been reduced significantly if surgery had been done.

## Conclusion

IE in the region occurs in a relatively younger population, with RHD being the most common underlying heart disease, and advanced valve lesions were also noted. There is a high rate of culture-negative endocarditis, and as a result, clinical diagnosis supported by echocardiography is the main diagnosis tool, and the majority of patients were treated empirically. Gram-negative bacilli predominate in our patients. Morbidity and mortality were very high. The in-hospital mortality was higher in patients who recommended surgery as an intervention and in patients with myalgia clinical symptoms. Special attention should be given to microbiology services, as there is a high culture-negative result. The establishment of cardiac surgery services should be given top priority, as it may reduce the mortality.

## Supporting information

S1 File (DTA)
